# Causality between the built environment and subjective wellbeing: Applying difference-in-differences and synthetic control methods to longitudinal data from England

**DOI:** 10.23889/ijpds.v8i2.2338

**Published:** 2023-09-14

**Authors:** Jerry Chen, Li Wan

**Affiliations:** 1 University of Cambridge, Cambridge, United Kingdom

## Objectives

Potential causal relationship between the built environment and subjective wellbeing has been segmentally explored and partially quantified. We leverage household relocation as a natural experiment to investigate the causality between built environment change and subjective wellbeing.

## Methods

Two causal inference methods (difference-in-differences and synthetic control) are applied and compared. The use of the ‘Understanding Society’ dataset (The UK Household Longitudinal Study, 2009-2019), combined with holistic locational attributes (Area Classification at the Lower Super Output Area level as per the UK Census) for exploring such causality is novel in literature. Specifically, to estimate the effects of relocation, we compare movers (treatment n=773) to non-movers (control n=4,619). To estimate the effects of built environment change, we compare movers with a change in built environment (n=506) with those moving to the same built environment type (n=267).

## Results

Our results show immediate and enduring positive causal effects of relocation, equivalent to an average improvement of 8% in subjective wellbeing level compared to non-movers. Among moves, moving to a different built environment improves subjective wellbeing by an equivalent of 13% compared with moving to the same built environment type. Without a change in built environment type, the positive causal effects become negligible. We further find the distress of relocation is transitory, and preliminary evidence that relocation decisions are formed over years and influenced by acute stressors. We hypothesise that relocation and change in built environment alleviate existing distresses but play limited roles in delivering multi-dimensional subjective wellbeing benefits.

## Conclusion

This paper is one of the first studies that apply and compare two causal models (DiD and SCM) for identifying potential causal effects of built environment on subject wellbeing. It is demonstrated that recent developments in causal inference methods have untapped potential to be applied in urban planning research. The ability to robustly identify complex causal/associative effects are particularly pertinent for policymaking purposes.

